# Dynamics from Seconds to Hours in Hodgkin-Huxley Model with Time-Dependent Ion Concentrations and Buffer Reservoirs

**DOI:** 10.1371/journal.pcbi.1003941

**Published:** 2014-12-04

**Authors:** Niklas Hübel, Markus A. Dahlem

**Affiliations:** 1 Department of Theoretical Physics, Technische Universität Berlin, Berlin, Germany; 2 Department of Physics, Humboldt Universität zu Berlin, Berlin, Germany; University of Pittsburgh, United States of America

## Abstract

The classical Hodgkin-Huxley (HH) model neglects the time-dependence of ion concentrations in spiking dynamics. The dynamics is therefore limited to a time scale of milliseconds, which is determined by the membrane capacitance multiplied by the resistance of the ion channels, and by the gating time constants. We study slow dynamics in an extended HH framework that includes time-dependent ion concentrations, pumps, and buffers. Fluxes across the neuronal membrane change intra- and extracellular ion concentrations, whereby the latter can also change through contact to reservoirs in the surroundings. Ion gain and loss of the system is identified as a bifurcation parameter whose essential importance was not realized in earlier studies. Our systematic study of the bifurcation structure and thus the phase space structure helps to understand activation and inhibition of a new excitability in ion homeostasis which emerges in such extended models. Also modulatory mechanisms that regulate the spiking rate can be explained by bifurcations. The dynamics on three distinct slow times scales is determined by the cell volume-to-surface-area ratio and the membrane permeability (seconds), the buffer time constants (tens of seconds), and the slower backward buffering (minutes to hours). The modulatory dynamics and the newly emerging excitable dynamics corresponds to pathological conditions observed in epileptiform burst activity, and spreading depression in migraine aura and stroke, respectively.

## Author Summary

The classical theory by Hodgkin and Huxley (HH) describes nerve impulses (spikes) that manifest communication between nerve cells. The underlying mechanism of a single spike is excitability, i.e., a small disturbance triggers a large excursion that reverts without further input to the original state. A spike lasts a 1/1000 second and, even though during this period ions are exchanged across the nerve cell membrane, the change in the corresponding ion concentrations can become significant only in series of such spikes. Under certain pathological conditions, changes in ion concentrations become massive and last minutes to hours before they recover. This establishes a new type of excitability underlying communication failure between nerve cells during migraine and stroke. To clarify this mechanism and to recognize the relevant factors that determine the slow time scales of ion changes, we use an extended version of the classical HH theory. We identify one variable of particular importance, the potassium ion gain or loss through some reservoirs provided by the nerve cell surroundings. We suggest to describe the new excitability as a sequence of two fast processes with constant total ion content separated by two slow processes of ion clearance (loss) and re-uptake (gain).

## Introduction

In this paper we study ion dynamics in ion-based neuron models. In comparison to classical HH type membrane models this introduces dynamics on much slower time scales. While spiking activity is in the order of milliseconds, the time scales of ion dynamics range from seconds to minutes and even hours depending on the process (transmembrane fluxes, glial buffering, backward buffering). The slow dynamics leads to new phenomena. Slow burst modulation as in seizure-like activity (SLA) emerges from moderate changes in the ion concentrations. Phase space excursions with large changes in the ionic variables establish a new type of ionic excitability as observed in cortical spreading depression (SD) during stroke and in migraine with aura [1], [2]. Such newly emerging dynamics can be understood from the phase space structure of the ion-based models.

Mathematical models of neural ion dynamics can be divided into two classes. On the one hand the discovery of SD by Leão in 1944 [3]—a severe perturbation of neural ion homeostasis associated with huge changes in the potassium, sodium and chloride ion concentrations in the extracellular space (ECS) [4] that spreads through the tissue—has attracted many modelling approaches dealing with the propagation of large ion concentration variations in tissue. In 1963 Grafstein described spatial potassium dynamics during SD in a reaction-diffusion framework with a phenomenological cubic rate function for the local potassium release by the neurons [5]. Reshodko and Burés proposed an even simpler cellular automata model for SD propagation [6]. In 1978 Tuckwell and Miura developed a SD model that is amenable to a more direct interpretation in terms of biophysical quantities [7]. It contains ion movements across the neural membrane and ion diffusion in the ECS. In more recent studies Dahlem et al. suggested certain refinements of the spatial coupling mechanisms, e.g., the inclusion of nonlocal and time-delayed feedback terms to explain very specific patterns of SD propagation in pathological situations like migraine with aura and stroke [8], [9].

On the other hand single cell ion dynamics were studied in HH-like membrane models that were extended to include ion changes in the intracellular space (ICS) and the ECS since the 1980s. While the first extensions of this type were developed for cardiac cells by DiFranceso and Noble [10], [11], the first cortical model in this spirit was developed by Kager, Wadman and Somjen (KWS) [12] only in 2000. Their model contains abundant physiological detail in terms of morphology and ion channels, and was in fact designed for seizure-like activity (SLA) and local SD dynamics. It succeeded spectacularly in reproducing the experimentally known phenomenology. An even more detailed model was proposed by Shapiro at the same time [13] who—like Yao, Huang and Miura for KWS [14]—also investigated SD propagation with a spatial continuum ansatz. Another model of SD investigated Ca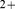 transmission along an astrocyte lane [15], where glutamate released from neurons that acts on metabotropic receptors of astrocytes determines the characteristics.

HH-like models of intermediate complexity were developed by Fröhlich, Bazhenov et al. to describe potassium dynamics during epileptiform bursting [16]–[18]. The simplest HH-like model of cortical ion dynamics was developed by Barreto, Cressman et al. [19]–[21] who describe the effect of ion dynamics in epileptiform bursting modulation in a single compartment model that is based on the classical HH ion channels. Interestingly, in none of these models, which are considerably simpler than, for example, Shapiro's model and the KWS model, extreme ion dynamics like in SD or stroke was studied. To our knowledge the only exception is a study by Zandt et al. who describe in the framework of Cressman et al. what they call the “wave of death” that follows the anoxic depolarization after decapitation as measured in experiments with rats [22].

In this study we systematically analyze the entire phase space of such local ion-based neuron models containing the full dynamical repertoire ranging from fast action potentials to slow changes in ion concentrations. We start with the simplest possible model for SD dynamics—a variation of the Barreto, Cressman et al. model—and reproduce most of the results for the KWS model. Our analysis covers SLA and SD.

**Figure 1:**
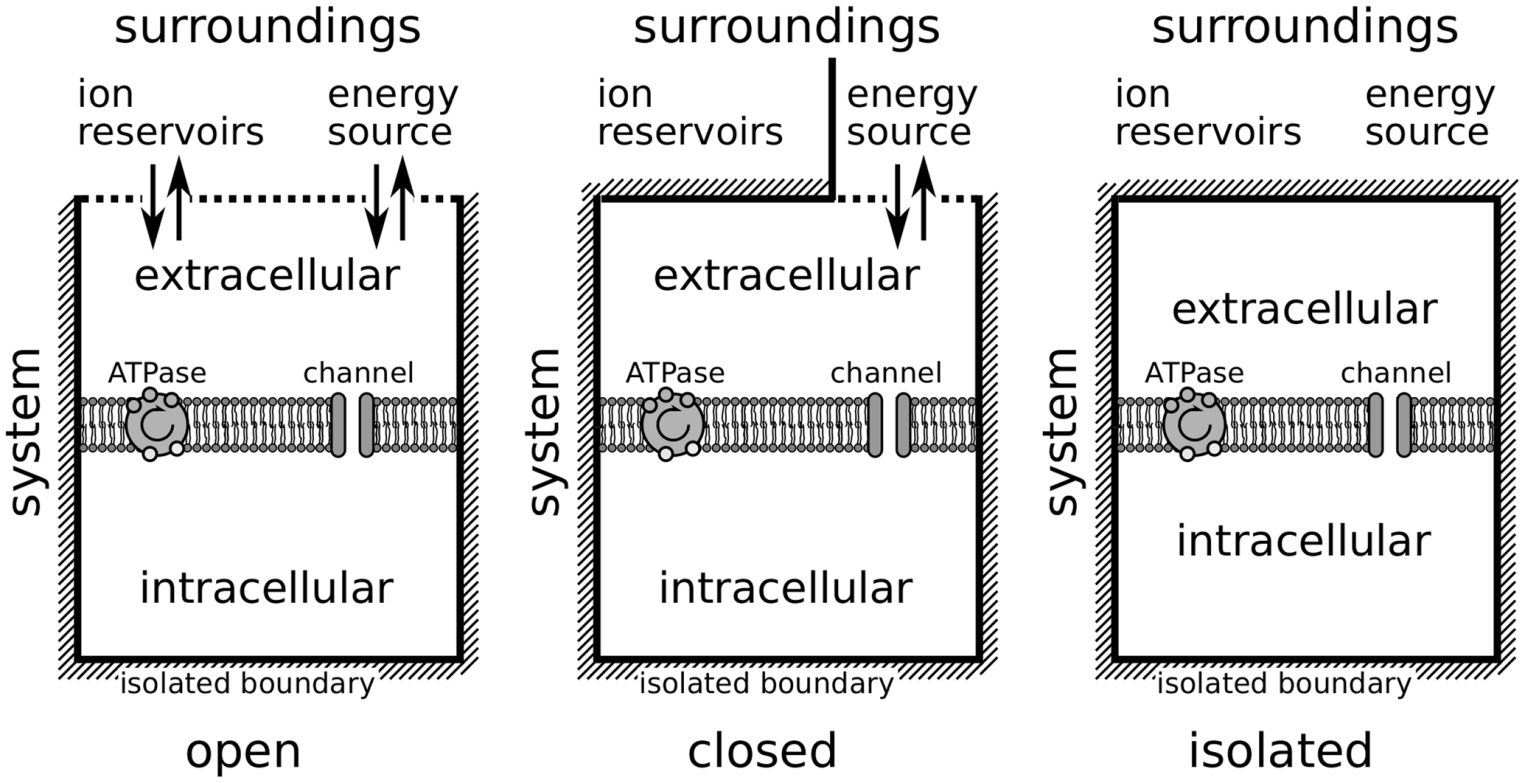
Neural tissue as a composite system with walls and surroundings. The ion–based model describes a system, comprising extracellular and intracellular compartments separated by a membrane, and the surroundings of the system. The latter provides an energy source and, if the system is not closed, also an ion reservoir.

Three situations should be distinguished: isolated, closed, and open systems, which is reminiscent of a thermodynamic viewpoint (see Fig. 1). An isolated system without transfer of metabolic energy for the ATPase-driven 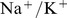 pumps will attain its thermodynamic equilibrium, i.e., its Donnan equilibrium. A closed neuron system with functioning pumps but without ion regulation by glial cells or the vascular system is generally bistable [23]. There is a stable state of free energy-starvation (FES) that is close to the Donnan equilibrium and coexists with the physiological resting state. The ion pumps cannot recover the physiological resting state from FES.

We will now develop a novel phase space perspective on the dynamics in open neuron systems. We describe the first slow-fast decomposition of local SD dynamics, in which the ion gain and loss through external reservoirs is identified as the crucial quantity whose essential importance was not realized in earlier studies. Treating this slow variable as a parameter allows us to derive thresholds for SD ignition and the abrupt, subsequent repolarization of the membrane in a bifurcation analysis for the first time. Moreover we analyze oscillatory dynamics in open systems and thereby relate SLA and SD to different so-called torus bifurcations. This categorizes SLA and SD as genuinely different though they are ‘sibling’ dynamics as they both bifurcate from the same ‘parent’ limit cycle in a supercritical and subcritical manner, respectively, which also explains the all-or-none nature of SD. In contrast, SLA is gradual.

## Model

Local ion dynamics of neurons has been studied in models of various complexity. Reduced model types consist of an electrically excitable membrane containing gated ion channels and ion concentrations in an intra- and an extracellular compartment [19]–[22]. Transmembrane currents must be converted to ion fluxes that lead to changes in the compartmental ion concentrations. Such an extension requires ion pumps to prevent the differences between ICS and ECS ion concentrations that are present under physiological resting conditions from depleting.

We consider a model containing sodium, potassium and chloride ions. The simulation code is available from ModelDB [24], the accession number is 167714. The HH-like membrane dynamics is described by the membrane potential 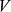 and the potassium activation variable 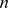. The sodium activation 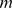 is approximated adiabatically and the sodium inactivation 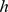 follows from an assumed functional relation between 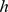 and 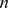. The ICS and ECS concentrations of sodium, potassium and chloride ions are denoted by 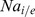, 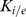 and 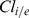, respectively.

In a closed system mass conservation holds, i.e., 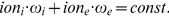
(1)with 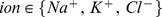 and the ICS/ECS volumes 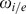. Together with the electroneutrality of ion fluxes across the membrane, i.e., 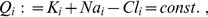
(2)only two of the six ion concentrations are independent dynamical variables. The full list of rate equations then reads 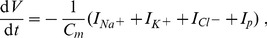
(3)

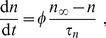
(4)

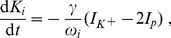
(5)

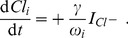
(6)


They are complemented by six constraints on gating variables and ion concentrations: 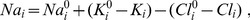
(7)

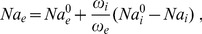
(8)

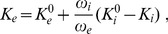
(9)

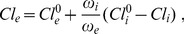
(10)

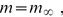
(11)

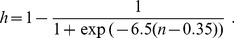
(12)


Superscript 0 indicates ion concentrations in the physiological resting state. Unless otherwise stated 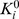 and 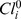 are used as initial conditions in the simulations. Constrained ion concentrations (Eqs. (7)–(10)) then also take their physiological resting state values. These ion concentrations, the membrane capacitance 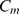, the gating time scale parameter 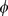, the conversion factor 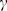 from currents to ion fluxes, and the ICS and ECS volumes 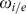 are listed in Tab. 1. The conversion factor 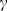 is an expression of the membrane surface area 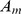 and Faraday's constant 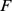 (both given in Tab. 1, too): 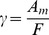
(13)We remark that all parameters in Tab. 1 are given in typical units of the respective quantities. The numerical values in these units can directly be used for simulations. Time is then given in msec, the membrane potential in mV and ion concentrations in 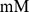.

**Table 1:** Parameters for ion–based model.

Name	Value & unit	Description
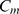	1 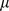 F/cm 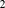	membrane capacitance
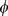	3/msec	gating time scale parameter
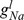	0.0175 mS/cm 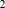	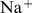 leak cond.
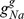	100 mS/cm 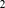	max. gated 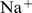 cond.
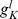	0.05 mS/cm 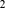	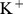 leak cond.
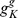	40 mS/cm 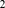	max. gated 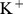 cond.
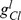	0.02 mS/cm 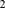	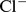 leak cond.
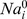	25.23 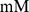	initial ICS 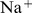 conc.
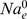	125.31 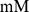	initial ECS 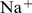 conc.
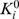	129.26 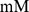	initial ICS 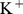 conc.
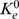	4 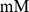	initial ECS 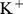 conc.
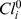	9.9 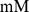	initial ICS 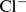 conc.
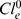	123.27 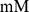	initial ECS 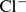 conc.
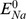	39.74 mV	initial 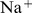 Nernst potential
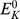	−92.94 mV	initial 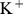 Nernst potential
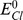	−68 mV	initial 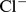 Nernst potential
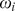	2,160 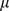 m 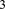	ICS volume
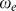	720 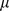 m 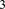	ECS volume
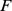	96485 C/mol	Faraday's constant
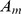	922 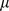 m 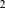	membrane surface area
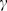	9.556e–2 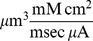	conversion factor
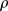	6.8 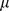 A/cm 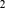	max. pump current
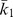	5e–5/ 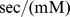	buffering rate
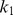	5e–5/sec	backward buffering rate
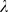	3e–2/sec	diffusive coupling strength
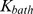	4 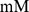	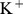 conc. of extracell. bath
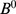	500 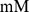	initial buffer conc.

The electroneutrality of the total transmembrane ion flux as expressed in Eqs. (2) and (7) is a consequence of the large time scale separation between the membrane dynamics and the ion dynamics (cf. Ref. [23] and the below discussion of time scales). This constraint is the reason why the thermodynamic equilibrium of the system must be understood as a Donnan equilibrium. This is the electrochemical equilibrium of a system with a membrane that is impermeable to some charged particles, which can be reached in an electroneutral fashion, i.e., without separating charges. We do not include this impermeant matter explicitly, because it does not influence the dynamics as long as osmosis is not considered. One should however keep in mind that the initial ion concentrations in Tab. 1 do not imply zero charge in the ICS or ECS and hence impermeant matter to compensate for this must be present.

The gating functions 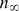, 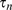 and 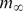 are given by 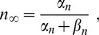
(14)

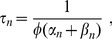
(15)

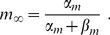
(16)Here 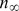 and 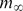 are the asymptotic values and 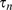 is potassium activation time scale. They are expressed in terms of the Hodgkin-Huxley exponential functions [19]–[21]

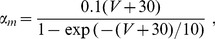
(17)

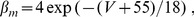
(18)

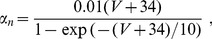
(19)

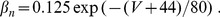
(20)


The three ion currents are 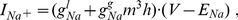
(21)

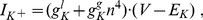
(22)

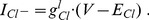
(23)They are given in terms of the leak and gated conductances 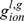 (with 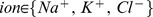) and the Nernst potentials 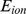 which are computed from the (dynamical) ion concentrations 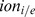: 
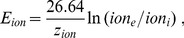
(24)

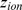
 denotes the valence of the particular ion species.

The pump current modelling the ATPase-driven exchange of intracellular sodium with extracellular potassium at a 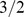-ratio is given by 
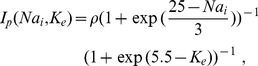
(25)where 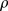 is the maximal pump rate [21]. The pump current increases with 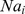 and 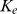. The values for the conductances and pump rate are also given in Tab. 1. Let us remark that in comparisons with Ref. [23], we have mildly increased the maximal pump rate and decreased the chloride conductance to obtain a SD threshold in agreement with experiments (see Sect. Results).

Eqs. (3)–(12) describe a closed system in which ion pumps are the only mechanism maintaining ion homeostasis and in which mass conservation holds for each ion species. A remark on terminology is due at this point: a ‘closed’ system refers exclusively to the conservation of the ion species that we model. We do not directly model other mass transfer that occurs in real neural systems. Yet it is indirectly included. The ion pumps use energy released by hydrolysis of ATP, a molecule whose components (glucose and oxygen or lactate) therefore have to pass the system boundaries. In thermodynamics, it is customary to call systems that exchange energy but not matter with their environment closed. Since ATP is in this framework only considered as an energy source, we can describe the system as closed, if ions cannot be transferred across its boundaries.

As mentioned above the closed system is bistable. Superthreshold stimulations cause a transition from physiological resting conditions to FES. To resolve this and change the behaviour to local SD dynamics it is necessary to include further regulation mechanisms [23]. Since SD is in particular characterized by an extreme elevation of potassium in the ECS we will only discuss potassium regulation.

If ECS potassium ions are subject to a regulation mechanism which is independent of the membrane dynamics, then the symmetry between ICS and ECS potassium dynamics is broken and Eq. (9) for the potassium conservation does not hold. Let us represent changes of the potassium content of the system by a variable 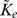 which is defined by the following relation: 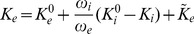
(26)


Changes of the potassium content, i.e., changes of 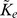, can be of different physiological origin. If glial buffering is at work the potassium content will be reduced by the amount of buffered potassium 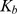. An initial potassium elevation 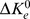 simply leads to an accordingly increased 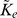: 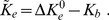
(27)For the coupling to an extracellular potassium bath or to the vasculature 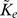 is a measure for the amount of potassium that has diffused into (positive 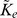) or out of (negative 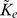) the system.

We are going to discuss two regulation schemes—coupling to an extracellular bath and glial buffering. They could be implemented simultaneously, but for our purpose it will suffice to apply only one scheme at a time. In the second subsection of Sect. Results, the dynamics of 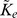 is given by glial buffering, while in the third subsection we will discuss the oscillatory regimes one finds for bath coupling with elevated bath concentrations. To implement glial buffering we assume a phenomenological chemical reaction of the following type [12], [25]: 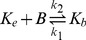
(28)The buffer concentration is denoted by 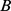. We are using the buffer model from Ref. [12] in which the potassium-dependent buffering rate 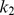 is given as 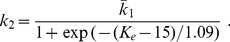
(29)The parameter 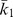 is normally assumed to have the same numerical value as the constant backward buffering rate 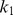 which is hence an overall parameter for the buffering strength. However, the parameters should be denoted differently as they have different units (cf. Tab. 1). This chemical reaction scheme together with the mass conservation constraint 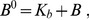
(30)where 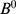 is the initial buffer concentration, leads to the following differential equation for 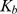: 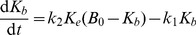
(31)
Eq. (27) the implies the following rate equation for 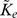


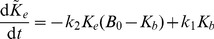
(32)where 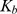 and 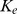 are given by Eqs. (27) and (26), respectively.

To model the coupling to a potassium bath one normally includes an explicit rate equation for the ECS potassium concentration 
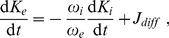
(33)where the diffusive coupling flux 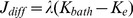
(34)is defined by its coupling strength 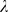 and the potassium bath concentration 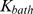. Eq. (26) implies that Eq. (33) can be rewritten in terms of 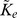 as follows: 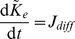
(35)Note that we have chosen to formulate ion regulation in terms of 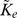 rather than 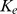 which would be completely equivalent. This is crucial, because the dynamics of 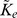 happens on a time scale that is only defined by the buffering or the diffusive process, while 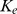 dynamics involves transmembrane fluxes and reservoir coupling dynamics at different time scales (cf. the last paragraph of this section). This can be seen from Eq. (33).

Both regulation schemes—glial buffering given by Eq. (32) and coupling to a bath with a physiological bath concentration as in Eq. (35)—can be used to change the system dynamics from bistable to ionically excitable, i.e., excitable with large excursions in the ionic variables. Like all other system parameters the regulation parameters 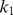 and 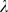 are given in Tab. 1. They have been adjusted so that the duration of the depolarized phase is in agreement with experimental data on spreading depression.

Note that the parameters we have chosen are up to almost one order of magnitude lower than intact brain values like the ones used in Refs. [12], [25]–[27]. While this does not affect the general time scale separation between glial or vascular ion regulation and ion fluxes across the cellular membrane, the duration of SD depends crucially on these parameters. However, during SD oxygen deprivation will weaken glial buffering, and the swelling of glial cells and blood vessel constriction will restrict diffusion to the vasculature. Such processes can be included to ion-based neuron models and make ion regulation during SD much slower [12], [25]–[27].

For our purpose it is however sufficient to assume smaller values from the beginning. We remark that the ion regulation schemes in our model only refer to vascular coupling and glial buffering. Lateral ion movement between the ECS of nearby neurons is a different diffusive process that determines the velocity of a travelling SD wave in tissue. This is not described in our framework. In the following section we will demonstrate in detail how 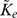 can be understood as the inhibitory variable of this excitation process.

The above presented model is indeed the simplest ion-based neuron model that exhibits local SD dynamics. Model simplicity is an appealing feature in its own right, but one might doubt the physiological relevance of such a reduced model. Our hypothesis is that it captures very general dynamical features of neuronal ion dynamics, and to confirm this we will compare the results obtained with the reduced model to the physiologically much more detailed KWS model [12]. This detailed model contains five different gated ion channels (transient and persistent sodium, delayed rectifier and transient potassium, and NMDA receptor gated currents) and has been used intensively to study SD and SLA. In fact, one modification is required so that we can replicate the results obtained from the reduced model. The KWS model contains an unphysical so-called ‘fixed leak’ current 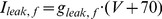
(36)that has a constant reversal potential of 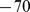 mV and no associated ion species. This current only enters the rate equation for the membrane potential 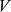.

The effect on the model dynamics is dramatic. To see this note that the electroneutrality constraint Eq. (8) reflects a model degeneracy
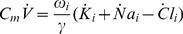
(37)that occurs when 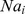 is modelled explicitly with 
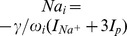
 (for details see Ref. [23]). With a fixed leak current Eq. (37) becomes 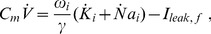
(38)which implies that 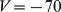 mV is a necessary fixed point condition for the system.

In other words, the type of bistability with a second depolarized fixed point that we normally find in closed systems is ruled out by this unphysical current. If we, however, replace it with a chloride leak current as in our model (cf. Eqs. (6) and (23)), i.e., a current with a dynamically adjusting reversal potential by virtue of Eq. (24), we find the same type of bistability for the closed system and monostability for the system that is buffered or coupled to a potassium bath. The morphological parameters (compartmental volumes 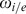 and membrane surface area 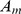) are the same as for the reduced model.

In fact in Ref. [14] the KWS model was used without additional ion regulation for a reaction-diffusion study of SD, and the only recovery mechanism of the local system seems to be this unphysical current. Theoretically SD could be a travelling wave in a reaction-diffusion system with bistable local dynamics, but unpublished results show that the propagation properties in the bistable system are dramatically different from standard SD dynamics with wave fronts and backs travelling at different velocities. We hence suppose that a local potassium clearing mechanism is crucially involved in SD.

We conclude this section with a discussion of the time scales of the model. To this end, it is helpful to keep in mind that the phenomenon of excitability requires a separation of time scales. We have electrical and ionic excitability and these dynamics themselves are separated by no fewer than three orders of magnitude.

Dynamics of 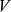 happens on a scale that is faster than milliseconds. This follows from the gating time scale 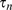 which is given explicitly in Eq. (15) and the time scale of 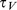 of 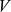 which can be computed from the membrane capacitance 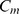 (given in Tab. 1) and the resistance 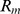 of the ion channels (for details see Ref. [28]): 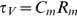
(39)with 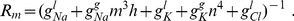
(40)If we approximate the products of gating variables in the above expression with 0.1 this gives 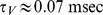. Dynamics of 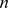 happens on a scale in the order of milliseconds.

The time scale of ion dynamics is more explicit in the Goldman-Hodgkin-Katz (GHK) formalism than in the Nernst formalism used in this paper. The Nernst currents in Eqs. (21)–(23) are an approximation of the physically more accurate GHK currents, but in Ref. [23] we have shown that ion dynamics of GHK models and Nernst models are very similar. That is why the latter may be used for studies like this. For time scale considerations, however, we will now switch to the GHK description. The GHK current of ions with concentrations 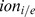 across a membrane is given by 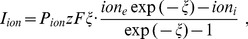
(41)where 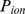 is the permeability of the membrane to the considered ion species and 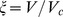 is the dimensionless membrane potential with 
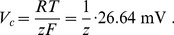
(42)This expression contains the ideal gas constant 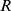, the temperature 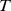, ion valence 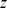 and Faraday's constant 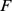. If we now write down the GHK analogue of the ion rate Eqs. (5) and (6) we obtain 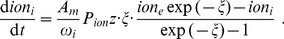
(43)For the conversion factor 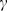 we have inserted the expression Eq. (13). The fraction term is of the order of the ion concentrations, 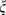 is a dimensionless quantity and hence of order one. With the ion dynamics time scale 
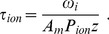
(44)we can thus group the parameters as follows 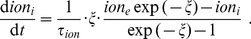
(45)Permeabilities of ion channels can be found in Refs [14], [23], [29]. Similar as for the resistance 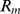 the permeability 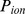 of a gated channel involves a product of gating variables. Approximating such terms again with 0.1 a typical value for the permeability is 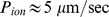. Together with the values for the membrane surface area and the cell volume from Tab. 1 the time scale of transmembrane ion dynamics is 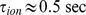.

The slowest time scales are related to potassium regulation, i.e., to 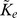 dynamics. The glia scheme from Eq. (28) and Eq. (32) contains a forward buffering process that reduces 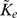 at a time scale 
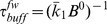
(46)and a backward buffering process with time scale 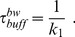
(47)With the parameters from Tab. 1 this leads to 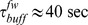 and 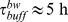. So backward buffering is much slower. This is an important property, because in the following section we will see that recovery from FES requires a strong reduction of the potassium content. If buffering and backward buffering would happen on the same time scale the required potassium reduction would not be possible. Backward buffering could well happen at a considerably faster scale than Eq. (47), but as soon as 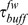 is comparable to 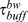 the buffer cannot re-establish physiological conditions after FES.

The glia scheme here is phenomenological. A more biophysically detailed model would describe a glial cell as a third compartment. An elevation of ECS potassium leads to glial uptake. Spatial buffering, i.e., the fast transfer of potassium ions between glial cells with elevated concentrations to regions of lower concentrations maintains an almost constant potassium level in the glial cells. In SD potassium in the ECS is strongly elevated during an about 80 sec lasting phase of FES and is continuously cleared during this time. After 80 sec the concentration quickly recoveres to even slightly less than the normal physiological level. Still there is a huge potassium deficit in the system and what we call backward buffering, i.e., the release of potassium from the glial cells, sets in. It is much slower than the uptake, because it is driven by a far smaller deviation of the potassium concentration from normal physiological resting level.

**Figure 2:**
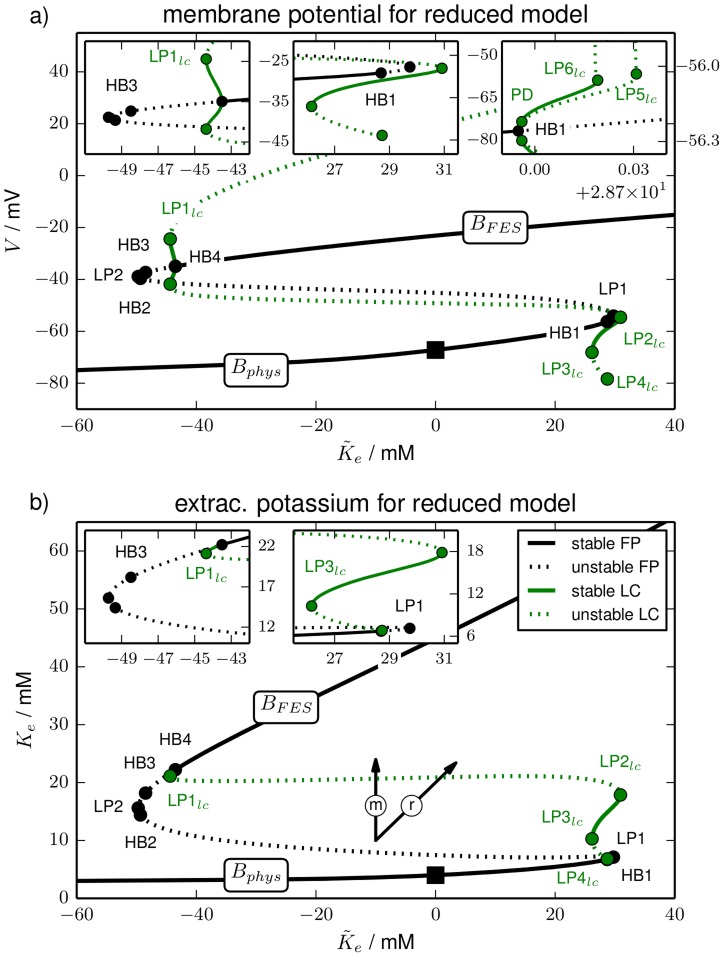
Bifurcation diagram. Bifurcation diagram of the reduced model for 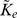 as the bifurcation parameter (purely transmembrane dynamics) showing (**a**) the membrane potential of fixed points (FP) and limit cycles (LC), and (**b**) potassium concentrations. The fixed point continuation yields the black curves. Solid sections are fully stable, dashed sections are unstable. The stability of the fixed point changes in HBs and LPs. The initial physiological condition is marked by a black square. The limit cycle is represented by the extremal values of the dynamical variables during one oscillation. The continuation yields the green lines with the same stability convention for solid and dashed sections. The stability of the limit cycle changes either in a LP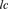 or in a period–doubling bifurcation (PD). In (**b**) the maximal and minimal extracellular potassium concentration of the limit cycle never differs by more than 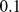 mM. The values can hence not be distinguished on the scale of this figure and therefore only the maximal value is drawn. The bifurcations are marked by full circles and labelled by the type, i.e., HB, LP or 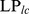, and a counter (cf. also the insets with blow–ups, in particular the rightmost one showing 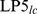 and 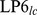 on a very small horizontal scale). The vertical and diagonal arrows labelled ‘m’ and ‘r’ indicate the direction of extracellular potassium changes due to ion fluxes across the membrane (‘m’) and changes only due to 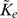, i.e., because of ion exchange with a reservoir (‘r’). Note that along the horizontal directions only the ICS potassium concentration changes by a precise mixture of fluxes across the membrane and ion exchange with a reservoir.

**Figure 3:**
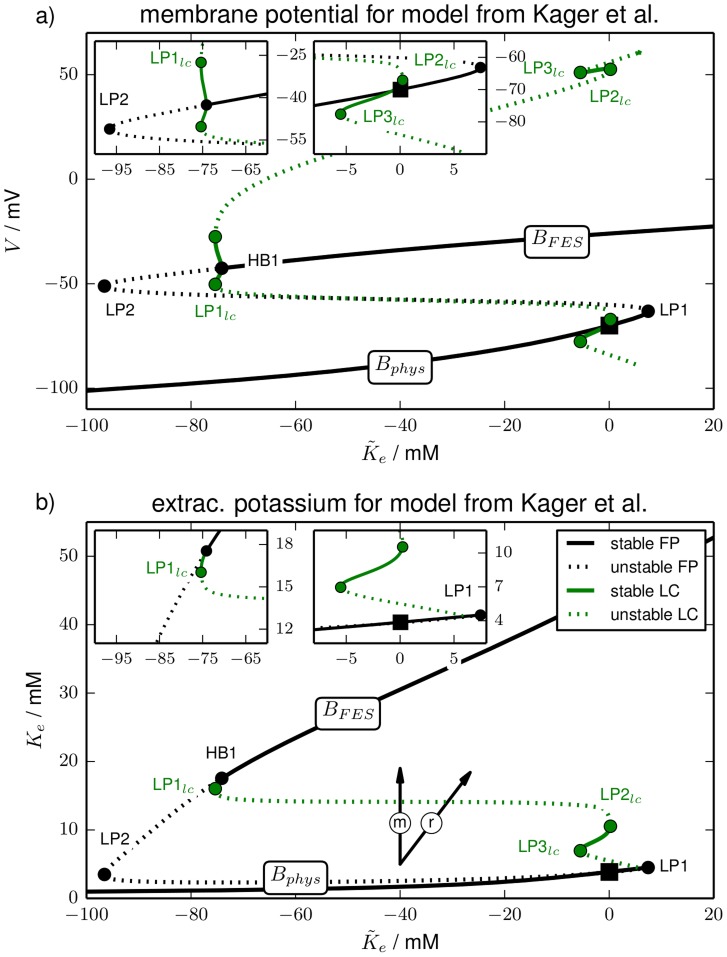
Bifurcation diagram. Bifurcation diagram of the model from Kager et al. (cf. last paragraph of Sect. Models). Like in Fig. 2 panel (**a**) shows the membrane potential and panel (**b**) shows the extracellular potassium concentration of the invariant sets, i.e., fixed points and limit cycles. The line style convention (solid for stable, dashed for unstable) and bifurcation labels are the same as in Fig. 2. Note the similar shape to Fig. 2, but also the different scale of the two figures.

Similar to the above explanation of slow backward buffering in the glia scheme, an extremely slow backward time scale follows naturally in diffusive coupling. For diffusion the potassium content is reduced at a time scale 
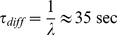
(48)if extracellular potassium is greater than 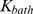. Backward diffusion, however, only occurs in the final recovery phase that sets in after the neuron has returned from the transient FES state and is repolarized. While 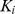 is still far from the resting state level, 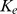 is comparable to normal physiological conditions (see the below bifurcation diagrams in Figs. 2b and 3b) and hence the driving force 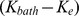 during the final recovery phase is very small for a bath concentration close to the resting state level. Consequently backward diffusion is much slower than forward diffusion.

Note that this argument for different slow regulation time scales relies exclusively on the almost constant values of the ECS potassium concentration along the physiological fixed point branch (see Figs. 2b and 3b). It is not a feature of the particular regulation scheme we apply.

## Results

The results are presented in three parts that describe (i) the stability of closed models, where we treat the change 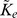 of the potassium content as a bifurcation parameter, (ii) open models, i.e., 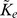 becomes a dynamical variable, with glial buffering and (iii) oscillations in ion concentrations in open models for bath coupling with the bath concentration 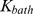 as a bifurcation parameter.

### Stability of closed models

At first we will not treat the change 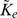 of the potassium content as a dynamical variable, but as a parameter whose influence on the system's stability we investigate. So the model we consider is defined by the rate Eqs. (3)–(6) and the constraint Eqs. (7), (8), (10)–(12) and (26). Its stability will be important for the full system with dynamical ion exchange between the neuron and a bath or glial reservoir to be discussed in the next two subsections. The phenomenon of ionic excitability as in SD only occurs for dynamical 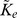. We will see that a slow-fast decomposition of ionic excitability is possible. The fast ion dynamics is governed by the transmembrane dynamics that we discuss now and happens at the time scale 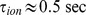. The dynamics of 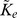 is much slower (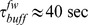 and 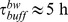). Fast ion dynamics of the full system can hence be understood by assuming 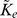 as a parameter that determines the level at which fast (transmembrane) ion dynamics occurs. This implies a direct physiological relevance of the closed system bifurcation structure with respect to potassium content variation for transition thresholds in the full (open) system.

The bifurcation diagram of the reduced model is presented in Fig. 2. It is shown in the 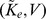-plane (Fig. 2a) and in the 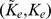-plane (Fig. 2b) to display membrane and ion dynamics, respectively. A pair of arrows pointing in the direction of extracellular potassium changes only due to fluxes across the membrane (vertical ‘m’ direction) and only due to exchange with a reservoir (diagonal ‘r’ direction) is added to Fig. 2b.

The fixed point continuation yields a branch (black line) where fully stable sections are solid and unstable sections are dashed. Stability changes occur in saddle-node bifurcations (also called limit point bifurcation, LP) and Hopf bifurcations (HB). In a LP the stability changes in one direction (zero-eigenvalue bifurcation), in a HB it changes in two directions and a limit cycle is created (complex eigenvalue bifurcation). A limit cycle is usually represented by the maximal and minimal value of the dynamical variables. However, the oscillation amplitude of the ionic variables is almost zero for the limit cycles in our model. Maximal and minimal values cannot be distinguished on the figure scale. Hence in the 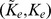-plane the limit cycle continuation appears only as a single line (green). Stability changes of limit cycles occur in saddle-node bifurcations of limit cycles (LP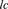). The limit cycles in our model disappear in homoclinic bifurcations. In this bifurcation a limit cycle collides with a saddle. When it reaches the saddle it becomes a homoclinic cycle of infinite period.

As a reference point the initial physiological condition is marked by a black square. We will call the entire stable fixed point branch that contains this point the physiological branch 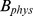, because the conditions are comparable to the normal functioning physiological state—in particular, action potential dynamics is possible when the system is on this branch.

Let us discuss the bifurcation diagram starting from this reference point and follow the fixed point curve in the right direction, i.e., for increasing 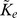. The physiological fixed point loses its stability in the first (supercritical) Hopf bifurcation (HB1) at 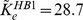 mM. The extracellular potassium concentration is then at 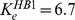 mM. In other word, much of the added potassium has been taken up by the cell.

The limit cycle associated with HB1 loses its stability in a period-doubling bifurcation (PD) and remains unstable. Finally it disappears in a homoclinic bifurcation shortly after its creation (cf. right inset in Fig. 2a). The stable limit cycle emanating from the PD point becomes unstable in a 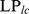 and vanishes in a homoclinic bifurcation, too. The parameter range of these bifurcations is extremely small (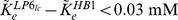). Such fine parameter scales will not play a role for the interpretation of ion dynamics. Ion concentrations are stationary and physiological up to 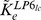, but for practical purposes it is irrelevant if we identify 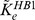 or 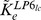 as the end of the physiological branch 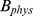.

The first HB is followed by four more bifurcations (LP1, HB2, LP2, HB3) that all neither restore the fixed point stability nor create any stable limit cycles. The limit cycles for HB2 and HB3 are hence not plotted either. It is only the fourth Hopf bifurcation (HB4) at 
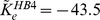
 mM in which the fixed point becomes stable again and in which a stable limit cycle is created. The limit cycle branch loses its stability in LP1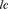 and regains it in LP2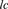. It becomes unstable again and even more unstable in LP3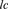 and LP4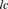. Shortly after that (not resolved on the scales in Fig. 2) it ends in a homoclinic bifurcation with the saddle between HB1 and LP2. At HB4 the stable free energy-starved branch 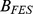 begins. It is generally characterized by a strong increase in the ECS potassium compared to physiological resting conditions (Fig. 2b), and a significant membrane depolarization (Fig. 2a). Corresponding to the extracellular elevation intracellular potassium is significantly lowered. This goes along with inverse changes of the compartmental sodium concentrations (all not shown). 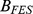 is hence characterized by largely reduced ion gradients and strong membrane depolarization. In fact, at this membrane potential the sodium channels are inactivated which is normally called depolarization block in HH-like membrane models without ion dynamics. Depolarization block is, however, only one feature of FES. The closeness of FES to the thermodynamic equilibrium of the system is more importantly manifested in the reduced ion gradients. On 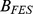 no more bifurcations occur and it remains stable for increasing 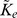.

The interpretation of this bifurcation diagram should be as follows. The end of 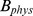 defines the maximal potassium content compatible with a physiological state of a neuron. For larger 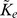 it will be inevitably driven to the FES. In other words the end of 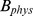 marks the threshold value for a slow, gradual elevation of the potassium content to cause the transition from physiological resting conditions to FES. In a buffered system it is the threshold for SD ignition. On the other hand stable FES-like conditions require a minimal potassium content which marks the end of 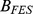. It is given by 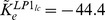 mM. Below this value the only stable fixed point is physiological. Again there is a narrow range, namely 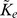 between 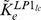 and 
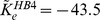
 mM, in which stable oscillations can occur.

When glial buffering is at work the end of 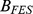 defines the threshold for potassium buffering, i.e., for the potassium reduction that is required to return from FES to physiological conditions (cf. Eq. (27)). In the second subsection of Sect. Results, we will see that this is exactly how ion regulation facilitates recovery in SD models.

There is another way the bifurcation diagram in Fig. 2b can be read. As we have remarked above the limit cycles of the model are characterized by large oscillation amplitudes in the membrane variables 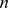 (not shown) and 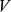, but almost constant ionic variables 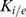, 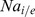 and 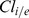 (only 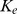 shown). So Fig. 2b tells us which extracellular potassium concentrations can possibly be stable and which ones cannot. Values below the end of 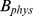 at 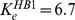 mM, values between 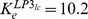 mM and 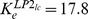 mM and finally concentrations in the range of 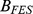 starting at 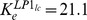 mM can be stable. Any other extracellular potassium concentration is unstable and the system will evolve towards a stable ion configuration that is present in the phase space. The highest stable potassium concentration below FES values is 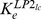. If potassium in the ECS is increased instantaneously, this value indicates the threshold for SD ignition or the transition to FES.

Performing the same type of bifurcation analysis with the physiologically more detailed model from Kager et al. [12], [14] (cf. last paragraph of Sect. Models) leads to the diagram in Fig. 3. It has been shown before that also in this model there is stable FES [23]. We do not find the same bifurcations as in the reduced model, but only two LPs and one HB. However, the physiological implications are very similar. Like in the reduced model there is an upper limit of the potassium content 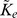 for stable physiological conditions (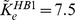 mM) and a lower limit for stable FES (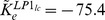 mM). Also the downward snaking and the stability changes of the limit cycle that starts from HB1 are very similar to Fig. 2. This leads to the same type of conclusion concerning possible stable extracellular potassium concentrations. While numerical values of the stability limits in terms of 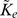 are specific to each model, the topological similarity of the bifurcation diagrams suggests a generality of results: there is a stable physiological branch 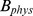 that ends at some maximal value 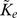 of the potassium content. Beyond this point the neuron cannot maintain physiological conditions, but will face FES. On the other hand the stable FES branch 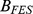 ends for a sufficiently reduced potassium content the neuron will return to physiological conditions.

The new bifurcation diagrams presented in this section confirm our results from Ref. [23]: Neuron models whose ionic homeostasis is only provided by ATPase-driven pumps, but without diffusive coupling or glial buffering, will have a highly unphysiological fixed point that is characterized by free energy-starvation and membrane depolarization. However, the presented bifurcation diagrams here contain additional information of great importance. Using the new bifurcation parameter 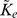 crucially extends our results from Ref. [23] by uncovering the threshold concentrations in extracellular potassium concentration. These are completely novel insights.

In the next subsection the bifurcation diagrams of the unbuffered (closed) systems shall facilitate a phase space understanding of the activation and inhibition process of ionic excitability as observed in SD in the buffered (open) systems. We are aiming for an interpretation of ionic excitability where neuronal discharge and recovery are fast dynamics that are governed by the bistable structure discussed above, whereas additional ion regulation takes the role of slowly changing 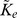.

However, only the gated ion dynamics, i.e., dynamics of sodium and potassium is fast compared to that of 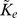, chloride is similarly slow. Due to the enforcement of electroneutrality this means that the overall concentration of positively charged ions in the ICS, i.e., the sum of sodium and potassium ion concentrations changes on the same slow time scale as the chloride concentration.

To describe this slow process not dynamically but—like 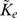
—in terms of a parameter we simply investigate the stability for a given distribution of non-dynamic, i.e., impermeant chloride. To determine this stability we set the chloride current to zero and vary 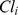 in a certain range (from 8 to 32 mM for the reduced model, and from 9 to 33 mM for the detailed model). This affects the system only through the electroneutrality constraint Eq. (7) which sets the intracellular charge concentration to be shared by sodium and potassium.

**Figure 4:**
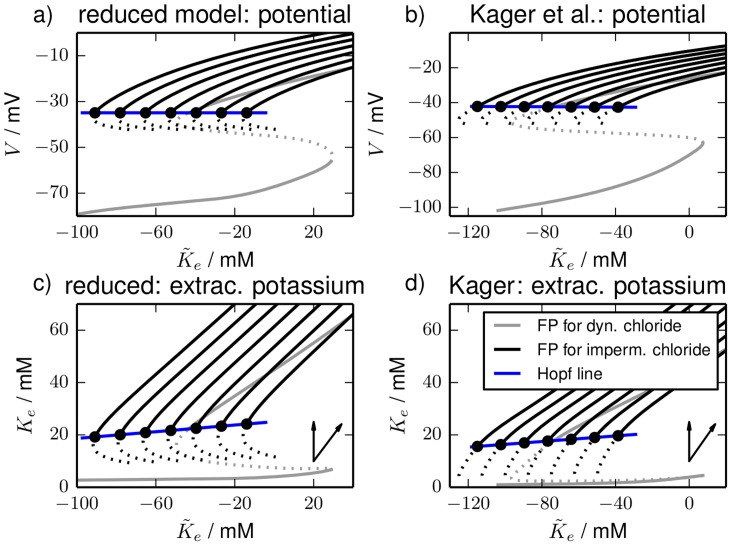
Fixed point continuation. Fixed point continuations for a range of impermeant intracellular chloride concentrations 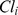 in (**a**), (**b**) the 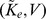
–plane and (**c**), (**d**) the 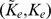
–plane. The black curves are the stable FES branches that lose their stability in Hopf bifurcations (black circles). Starting from the leftmost fixed point curves the fixed 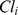 values are 8, 12, 16, 20, 24, 28 and 32 mM for the reduced model and 9, 13, 17, 21, 25, 29 and 33 mM for the detailed model. The Hopf bifurcations for different chloride concentrations lead to the blue Hopf line. As a reference the fixed point curves from Figs. 2 and 3 are also included in the diagram and drawn in grey.

For each value of 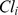 we perform a fixed point continuation as in Figs. 2 and 3 which yields similarly folded s-shaped curves. The result is shown in Fig. 4. For our analysis of SD it is only relevant where 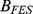 ends. That is why the plot does not contain the whole fixed point curve, but only 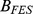 and a part of the unstable branch for a selection of 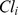 values. As a reference the diagrams also contain the fixed point curves from Figs. 2 and 3 which include chloride dynamics. The FES branches in Fig. 4 end in Hopf bifurcations. The bifurcation points for different chloride concentrations yield the blue Hopf line. It marks the threshold for recovery from FES when dynamics of chloride and 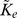 is slow.

### Open models with glial buffering

In the previous subsection we have analyzed the phase space structure of ion-based neuron models without contact to a reservoir, i.e., without glial buffering or diffusive coupling. These models have only transmembrane ion dynamics and obey mass conservation of each ion species. Hence they describe a closed system. The bistability of a physiological state and FES that we found in these closed models is not experimentally observed, because real neurons are always open systems not merely in the sense that they consume energy—a necessary prerequisite for being far from thermodynamic equilibrium—but they also can lose or gain ions through reservoirs or buffers. We will now include glial buffering and show how it facilitates recovery from FES, a condition which in contrast to the physiological state is close to a thermodynamic equilibrium, namely the Donnan equilibrium (cf. Ref. [23]).

When glial buffering is at work, 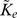 becomes a dynamical variable whose dynamics is given by the buffering rate Eq. (32). In a previous subsection we have explained that the bifurcation diagrams in Figs. 2 and 3 imply thresholds for an elevation of extracellular potassium to trigger the transition from physiological resting conditions to FES. This is in agreement with computational and experimental SD studies in which high extracellular potassium concentrations are often used to trigger SD. Another physiologically relevant way of SD ignition is the disturbance or temporary interruption of ion pump activity. As we have shown in Ref. [23] there is a minimal pump rate required for normal physiological conditions in a neuron. Below this rate the neuron will go into a FES state and remain in that state even when the pump activity is back to normal.

**Figure 5:**
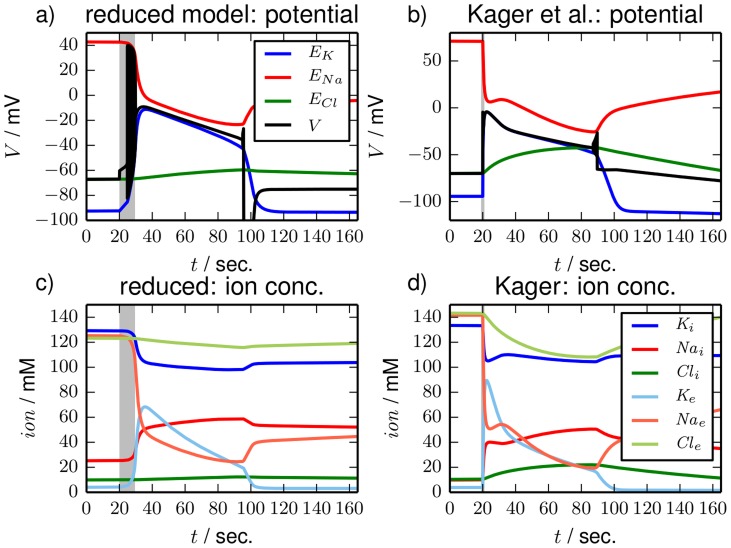
Time series. Time series for single SD excursions in (**a**), (**c**) the reduced and in (**b**), (**d**) the detailed model. In the reduced model SD is triggered by an interruption of the pump activity for about 10 sec (shaded region). In the detailed model the extracellular potassium concentration is increased by 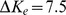 mM after 20 sec (vertical line). In (**a**) and (**b**) the time series of the membrane potentials (black lines) are shown. Nernst potentials for all ion species are included to the diagrams as a reference. Ion dynamics are shown in (**c**) and (**d**) where extracellular ion concentrations are in lighter color.

For the simulations in Fig. 5 we have interrupted the pump activity for about 10 sec in the reduced model, and we have elevated the extracellular potassium concentration by 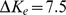 mM in the detailed model to trigger SD. Both stimulation types work for both models, but only the two examples are shown. The phase of pump interruption (Fig. 5a and 5c) is indicated by the shaded region in the plots, the time of potassium elevation is marked by the vertical grey line. The dynamics of the two models is very similar: in response to the stimulation the neuron strongly depolarizes and remains in that depolarized state for about 70 sec (Fig. 5a and 5b). After that the neurons repolarize abruptly and asymptotically return to their initial state. In addition to the membrane potential (black curve) the potential plots also contain the Nernst potentials for sodium (red line), potassium (blue line) and chloride (green line) that change with the ion concentrations according to the definition of the Nernst potentials in Eq. (24). In Fig. 5c and 4d we see that the potential dynamics goes along with great changes in the ion concentrations. In particular, extracellular potassium is strongly increased in the depolarized phase. These conditions are very similar to the type of FES states discussed in the previous subsection. The recovery of ion concentrations sets in with the abrupt repolarization, but it is a very slow asymptotic process that is not shown in Fig. 5.

In both models the neuron is capable of producing spiking activity again right after the repolarization. All these aspects of ion dynamics during SD are well-known from several studies [12], [14]. We remark that the time series are almost identical if glial buffering is replaced by the coupling to a potassium bath. Both, the strength of glial buffering and of diffusive coupling have been adjusted so that the depolarized phase lasts about 70 sec which is the experimentally determined time. We will focus on bath coupling in last subsection of Sec. Results. If neither buffering nor a potassium bath is included the neuron does not repolarize (for time series plots of terminal transitions to FES see Ref. [23]).

**Figure 6:**
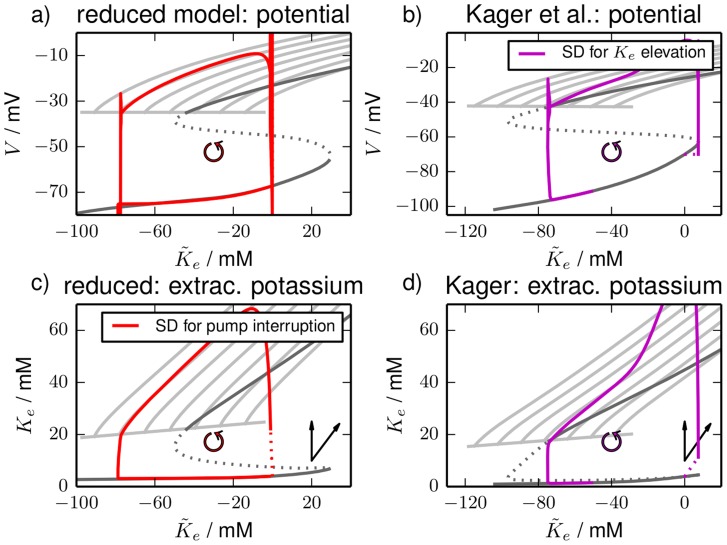
Phase space plots. Phase space plots of the simulations in Fig. 5. As in Fig. 4 panels (**a**) and (**b**) contain plots of the membrane potentials, in panels (**c**) and (**d**) extracellular potassium is shown. (**a**) and (**c**) are for the reduced model, (**b**) and (**d**) for the detailed model. The trajectories of the reduced model are represented as red curves, those of the detailed model are magenta. The sections of the trajectories that belong to times before and during the stimulation are dashed. The fixed point curves from Fig. 4 are added on the plots as shaded lines whereas the fixed point continuations for the unbuffered models with dynamical chloride are slightly darker. The pair of arrows in the extracellular potassium plots indicates the direction of pure transmembrane (vertical) and pure buffering dynamics (diagonal).

The time series in Fig. 5 are useful to confirm that the neuron models we investigate have the desired phenomenology and indeed show SD-like dynamics. Yet the nature of the different phases of this ionic excitation process—the fast depolarization, the prolonged FES phase and the abrupt repolarization—remains enigmatic [12], [14], [29], [30]. In a phase space plot the picture becomes much clearer and the entire process can be directly related to the two stable branches, 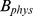 and 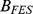, that we found for the closed and therefore pure transmembrane models in the previous subsection. In Fig. 6 the time series from Fig. 5 for a simulation time of 50 min are shown in the 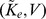
- and the 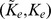-plane. The parts of the trajectories during the stimulation (pump interruption and potassium elevation) are dashed. In the chosen planes vertical lines belong to dynamics of constant potassium contents that can be understood in terms of the models we analyzed in the previous subsection. That is why Fig. 6 contains the fixed point curves from Fig. 4 as shaded lines as a guide to the eye. In Fig. 6c and 6d buffering dynamics is diagonal as indicated by the pair of arrows added to the plot.

For both trajectories the stimulation is followed by a vertical activation process that leads to the transition from 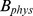 to 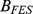. The verticality means that this is a process almost purely due to transmembrane dynamics. It is governed by the bistable phase space structure that we discussed in the previous section and also in Ref. [23]. Buffering dynamics is too slow to inhibit the activation. The types of stimulation we applied are related to bifurcations of the transmembrane system: the potassium elevation is beyond the end of 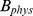 which is marked by the first Hopf bifurcation (HB1) in Fig. 2. The interruption of pump activity means that we go below a pump rate threshold that is defined by a saddle-node bifurcation (cf. Ref. [23]). More generally, to initiate an ionic excitation it is necessary to stimulate the system until it enters the basin of attraction—derived in the unbuffered system—of the FES state. The activation is followed by a phase of both, slow transient transmembrane dynamics mostly due to chloride, and potassium buffering. It is the latter that bends the trajectories in the diagonal direction so that they go along the FES branches from Fig. 4. The trajectories slowly approach the repolarization threshold given by the Hopf line. The duration of this FES phase is determined by how long it takes the system to reach the Hopf line.

This process is a mixture of buffering and transient transmembrane dynamics for the reduced model and more buffering-dominated in the detailed model. The duration of the FES phase is consequently a result of both types of dynamics. However, the main insight we gain from this plot is: glial buffering is the necessary inhibitory mechanism that takes the system to the Hopf line so that it can repolarize. We remark that the time series and phase space plots for bath coupling instead of buffering are almost identical and the same interpretation holds. The more general conclusion is then: ion dynamics beyond transmembrane processes is necessary to take the system to the Hopf line so that it can repolarize. This can, of course, be a combination of bath coupling and buffering. When the Hopf line is reached that neuron repolarizes abruptly which is the second almost purely vertical process. The repolarization is followed by slow asymptotic recovery dynamics of ion concentrations that takes the neuron back to the initial state which is at 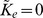 mM. The neuron regains the electrical excitability that is lost during FES already right after the repolarization. So the system is back to physiological function long before the ion gradients are fully restored.

Let us summarize the results from this subsection. By relating the SD time series from Fig. 5 to the bifurcation structure of the unbuffered models from the first subsection of Sect. Results and in particular to the two stable branches 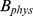 and 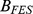 we have succeeded to understand ionic excitability as a sequence of different dynamical phases. The initial depolarization and the later repolarization are membrane-mediated fast processes that obey the bistable dynamics of unbuffered systems. The FES phase is buffering-dominated and lasts until buffering has taken the system to a well-defined repolarization threshold. The recovery phase is dominated by backward buffering. The full excursion time is the sum of the durations of each phase. For the de- and repolarization process this duration mainly depends on the time scale of the transmembrane dynamics and is hence comparably short. The duration of the FES phase is a result of both, the transient transmembrane dynamics and glial ion regulation at a much slower time scale. The final recovery phase is mainly backward buffering dominated which is the slowest process. Hence the duration of an SD excursion is mainly determined by the slow buffering and backward buffering time scales. This conclusion that relies on our novel understanding of the different thresholds involved in SD is in fact in agreement with recent experimental data suggesting vascular clearance of extracellular potassium as the central recovery mechanism in SD [31], [32].

### Ionic oscillations for bath coupling

The dynamics of excitable systems can often be changed to self-sustained oscillations by a suitable parameter variation. The type of bifurcation that leads to the oscillations and the shape of the limit cycle in the oscillatory regime determine excitation properties like threshold sharpness and latency [28]. The oscillatory dynamics that is related to ionic excitability can be obtained for bath coupling with an elevated bath concentration 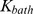. So in this section we replace the buffering dynamics for 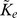 with the diffusive coupling given by Eq. (35). This coupling is used in experimental in-vitro studies of SD [33] and has also been applied in computational models that are very similar to our reduced one [19]–[21].

**Figure 7:**
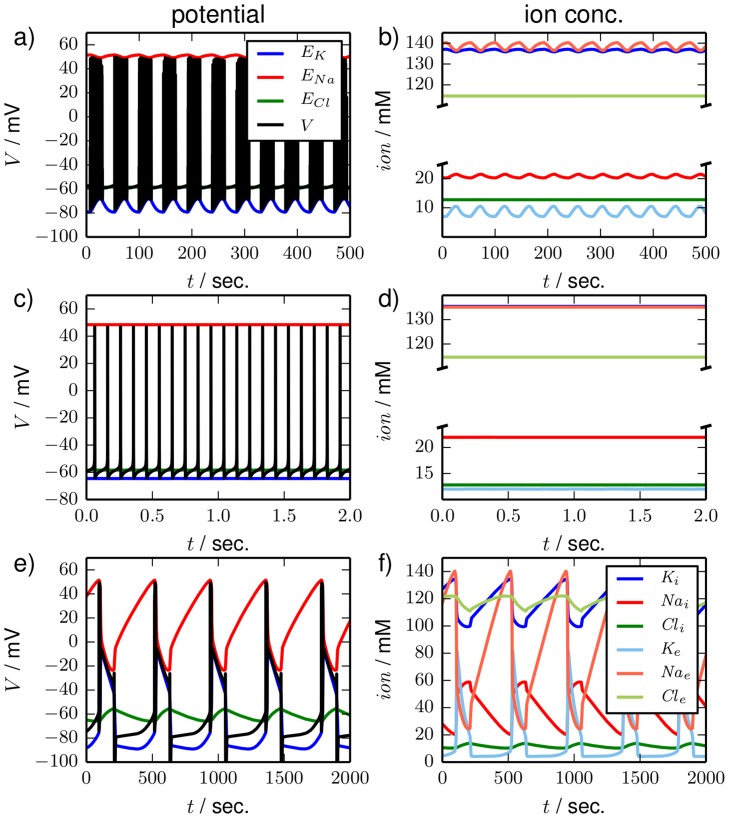
Time series. Time series for three types of oscillatory dynamics in the bath coupled reduced model. In the left panels (**a**), (**c**) and (**e**) the membrane potential and the three Nernst potentials are shown. Ion concentrations are shown in the right panels (**b**), (**d**) and (**f**). The color code is as in Fig. 5. (**a**) and (**b**), (**c**) and (**d**), and (**e**) and (**f**) are simulations for 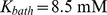, 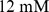 and 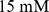, respectively. The dynamics is typical for (**a**) and (**b**) seizure–like activity, (**c**) and (**d**) tonic firing, (**e**) and (**f**) periodic SD. Note the different time scales of SLA, tonic firing and period SD and also the different oscillation amplitudes in the ionic variables.

Depending on the level of the bath concentration, we find three qualitatively different types of oscillatory dynamics that are shown in Fig. 7. The top row (a) shows the time series of seizure-like activity for 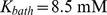. It is characterized by repetitive bursting and low amplitude ion oscillations. The other types of oscillatory dynamics are tonic firing at 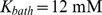 with almost constant ion concentrations (Fig. 7b) and periodic SD at 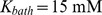 with large ionic amplitudes (Fig. 7c). We see that SLA and periodic SD exhibit slow oscillations of the ion concentrations and fast spiking activity, which hints at the toroidal nature of these dynamics. Below we will relate SLA and periodic SD to torus bifurcations of the tonic firing limit cycle.

The examples in Fig. 7 show that our model contains a variety of physiologically distinct and clinically important dynamical regimes. A great richness of oscillatory dynamics, in fact, under the simultaneous variation of 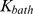 and the glial buffering strength has already been reported in Refs. [19]–[21] for a very similar model. In Ref. [19], [20] the authors even give a bifurcation analysis of ionic oscillations for 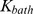 elevation.

To investigate dynamical changes and the transitions between the dynamical regimes in our model we perform a similar bifurcation analysis and vary 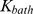, too. Two important differences should be noted though. First, Ref. [19], [20] uses an approximation of the multi-time scale model in which the fast spiking dynamics is averaged over time, while our analysis does not rely on such an approximation. Second, our analysis covers a bigger range of 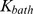 values which allows us to compare SLA and SD, while Ref. [19], [20] exclusively deals with SLA.

**Figure 8:**
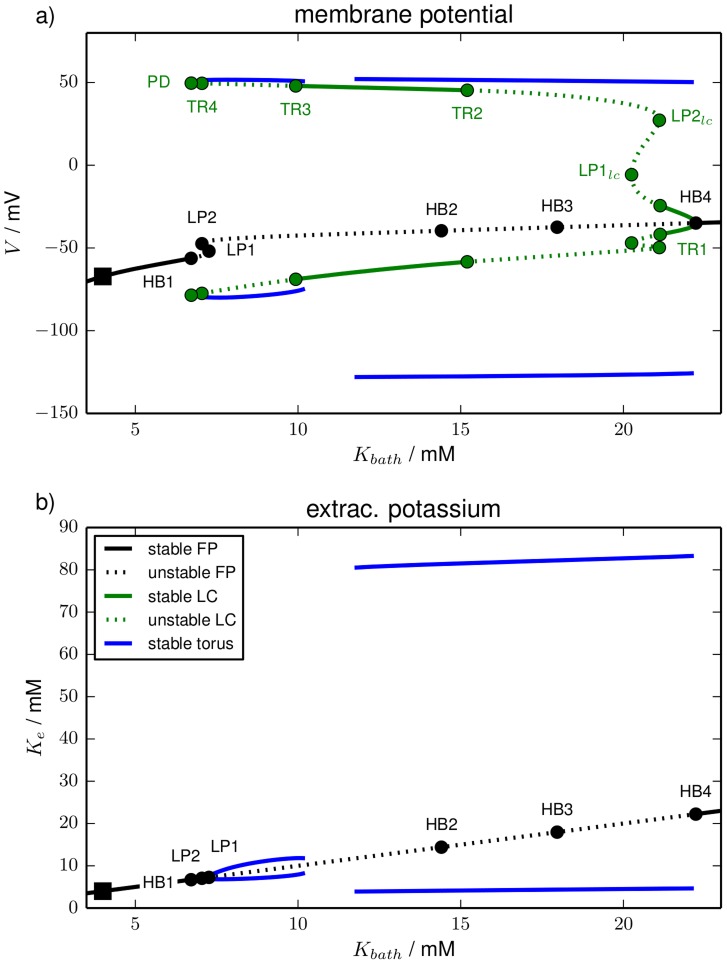
Bifurcation diagram. Bifurcation diagram of the bath coupled reduced model for 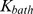
–variation. Color and line style conventions for fixed points and limit cycles are Figs. 2 and 3: black and green lines are fixed point and limit cycles, solid and dashed line styles mean stable and unstable sections. Stable solution on invariant tori are blue. They were obtained by direct simulations. The fixed point changes stability in HBs and LPs. The bifurcation types limit cycle undergoes are 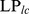, period–doubling (PD) and torus bifurcation (TR). Some physiologically irrelevant unstable limit cycles are omitted (cf. text). Panel (**a**) shows the membrane potential, panel (**b**) shows the extracellular potassium concentration. (**b**) does not contain the limit cycle, because it can hardly be distinguished from the fixed point line.

Fig. 8 shows the bifurcation diagram for 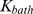 variation in the 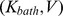-plane and in the 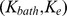-plane. In addition to fixed points (black) and limit cycles (green) also quasiperiodic torus solutions (blue) are contained in the diagram. In comparison to Fig. 2 this model contains a new type of bifurcation, namely a torus bifurcation (TR). A torus bifurcation is a secondary Hopf bifurcation of the radius of a limit cycle in which an invariant torus is created. If this torus is stable, nearby trajectories will be asymptotically bound to its surface. However, we cannot follow such solutions with standard continuation techniques, because these require an algebraic formulation in terms of the oscillation period. This is not possible for torus solutions, because on a torus the motion is quasiperiodic, i.e., characterized by two incommensurate frequencies. We can hence only track the stable solutions by integrating the equations of motion and slowly varying 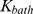. It is due to this numerically expensive method that in this section we will only analyze oscillatory dynamics of the reduced HH model with time-dependent ion concentrations.

The result of this bifurcation analysis in Fig. 8 shows us that there is a maximal level 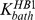 of the bath concentration compatible with physiological conditions. It is identified with the subcritical Hopf bifurcation HB1 in which the fixed point loses its stability. The related limit cycle is omitted, because it stays unstable and terminates in a homoclinic bifurcation with the unstable fixed point branch. The fixed point undergoes further bifurcations (LP1, LP2, HB2, HB3) which all leave it unstable and do not create stable limit cycles. It is in HB4 that the fixed point becomes stable again and also a stable limit cycle is created. This is the last fixed point bifurcation of the model.

The limit cycle that is created in HB4 changes its stability in several bifurcations. The physiologically most relevant ones are the four torus bifurcations. The bifurcation labels indicate the order of detection for the continuation that starts at HB4. Initially the limit cycle is characterized by fast low-amplitude oscillations. It becomes unstable in the subcritical torus bifurcation TR1. It regains and again loses its stability in the subcritical torus bifurcations TR2 and TR3. The last torus bifurcation, the restabilizing supercritical TR4, is directly followed by a PD after which no stable limit cycles exist any more. Again we have omitted in the diagram the unstable branch after PD and the limit cycle that is created in PD, which remains unstable.

Physiologically it is more intuitive to discuss the diagram for increasing 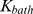 starting from the initial physiological conditions marked by the black square. Normal physiological conditions become unstable at 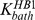 and above this value the neuron spikes continuously according to the stable limit cycle branch between PD and TR4. When 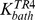 is reached the dynamics changes from stationary spiking to seizure-like activity on an invariant torus. The beginning of SLA is hence due to a supercritical torus bifurcation and the related ionic oscillation sets in with finite period and zero amplitude. From 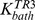 on tonic spiking activity is stable again and there is a small 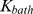-range of bistability between SLA and this tonic firing. As we mentioned above solutions on an invariant torus cannot be followed with normal continuation tools like AUTO, so only stable branches are detected. The details of the bifurcation scenario at TR3 are hence not totally clear, but we suspect that the unstable invariant torus that must exist near TR3 collides with the right end of the stable torus SLA-branch in a saddle-node bifurcation of tori. Tonic spiking then remains stable until TR2. This bifurcation is related to the period SD that already exist well below 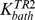. In fact, the threshold value 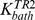 is in agreement with experiments [33]. Again the unstable torus near TR2 is not detected, but we suppose that a similar scenario as in TR3 occurs. The dynamics on the torus branch related to TR2 (and TR1 where it seems to end) is very different from the first torus branch. While the periods of the slow oscillations during SLA are 16–45 sec the ion oscillations of periodic SDs are much slower with periods of 350–550 sec.

Another crucial difference is obvious from Fig. 8b which shows the bifurcation diagram in the 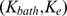-plane. The fixed point is just a straight line, because the diffusive coupling Eq. (35) makes 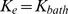 a necessary fixed point condition. The limit cycle is always extremely close to this line. On the chosen scale it cannot be distinguished from the fixed point and is hence not contained in the plot. Only the torus solutions of SD and SLA attain 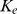 values that differ significantly from the regulation level. The ionic amplitudes of SD are one order of magnitude larger than those of SLA. This has to do with the fact that the peak of SD—as described above—must be understood as a metastable FES state that exists due to the bistability of the transmembrane dynamics. The dynamics of SLA is clearly of a different nature.

Note that the bifurcation diagram reveals a bistability of tonic firing and full-blown SD between the left end of the SD branch at about 11 mM and TR2. This means that there is no gradual increase in the ionic amplitudes that slowly leads to SD, but instead it implies that SD is a manifest all-or-none process.

**Figure 9:**
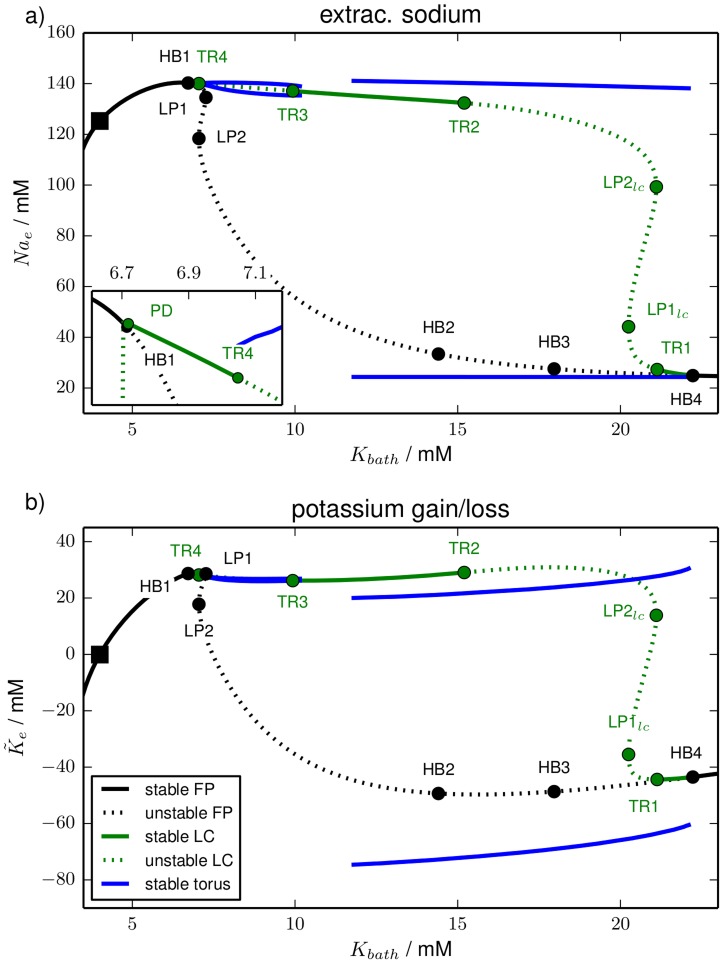
Bifurcation diagram. Different representations of the bifurcation diagram of Fig. 8. Panel (**a**) shows the extracellular sodium concentration and includes an inset around TR4 and PD. Panel (**b**) presents the potassium gain/loss.

In Fig. 9 we look at the same bifurcation diagram in the 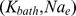
- and the 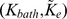-plane. While in Fig. 8 most of the ionic phase space structure is hidden, because 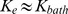 for fixed points and limit cycles, the 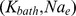-presentation in Fig. 9a provides further insights into the ion dynamics. We see that the stable fixed point branch before HB1 has extracellular sodium concentrations close to the physiological value 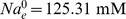. The stable branch after HB4, however, has an extremely reduced extracellular sodium level and is indeed FES-like. The stable limit cycles between PD and TR4 and between TR3 and TR2, and also SLA are rather close to the physiological sodium level. On the other hand, periodic SD is an oscillation between FES and normal physiological conditions, which is an expected confirmation of the findings from the previous section.

Fig. 9b is useful in connecting the phase space structure of the bath coupled system to that of the transmembrane model of the first subsection of Sect. Results. If we interchange the 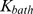
- and the 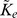-axis in the diagram it looks very similar to Fig. 2b. The torus bifurcations TR1, TR2 and TR3 are very close to the limit point bifurcations 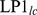, 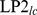 and 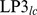 of the transmembrane model. The fixed point curves are topologically identical.

This striking similarity has to do with the fact that the limit cycle in Fig. 2 has almost constant ion concentrations. We have pointed out in the first subsection of Sect. Results that Fig. 2 tells us which extracellular potassium concentrations are stable for pure transmembrane dynamics. Diffusive coupling with bath concentrations at such potassium levels leads to negligibly small values of 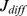 (cf. Eq. (35)). Therefore the limit cycle is still present in the bath coupled model and also the stability changes can be related to those in the transmembrane model. Again this can be seen as a confirmation of the results from the previous section: the transmembrane phase space plays a central role for models that are coupled to external reservoirs. We can interpret the ionic oscillations from Fig. 7 and the bifurcations leading to them with respect to this phase space.

**Figure 10:**
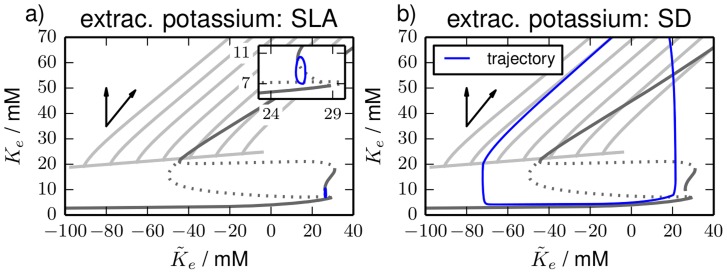
Phase space plots. Phase space plots of the simulations (**a**) for SLA and (**b**) periodic SDs from Fig. 7. Only extracellular potassium is shown. The limit cycle and fixed point curves from Figs. 2 and 4 are superimposed to the plots as shaded lines whereas the limit cycle and fixed point from Fig. 2 (dynamical chloride) are darker. The limit cycle and fixed point are not graphically distinguished, but comparison with Fig. 2 should avoid confusion.

Last we consider the dynamics of SLA and periodic SD in a phase space projection. In Fig. 10 the trajectories for SLA and periodic SD are plotted in the 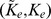-plane together with the underlying fixed point and limit cycles from the transmembrane model (cf. Fig. 4). The periodic SD trajectory has a very similar shape to the single SD excursion from Fig. 6 and is clearly guided by the stable fixed point branches 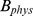 and 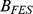. On the other hand SLA is a qualitatively very different phenomenon. Rather than relating to the FES branch, it is an oscillation between physiological conditions and those stable limit cycles that exist for moderately elevated extracellular potassium concentrations. The ion concentrations remain far from FES. So SLA and SD are not only related to distinct bifurcations, though of similar toroidal nature and branching from the same limit cycle, but they are also located far from each other in the phase space. This completes our phase space analysis of local ion dynamics in open neuron systems.

## Discussion

In this paper we have analyzed dynamics at different time scales in a HH model that includes time-dependent ion concentrations. Such models are also called second generation Hodgkin-Huxley models. They exhibit two types of excitability, electrical and ionic excitability, which are based on fast and slow dynamics. The time scales of these types of excitability are themselves separated by four to five orders of magnitude. The dynamics ranges from high-frequency bursts of about 100 Hz with short interburst periods of the order of 10 msec (Fig. 7a) to the slow periodic SD with frequencies of about 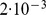 Hz and periods of about 7:30 min (Fig. 7c).

The slow SD dynamics in our model is classified as ultra-slow or near-DC (direct current) activity and cannot normally be observed by electroencephalography (EEG) recordings, because of artifacts due to the resistance of the dura (thick outermost layer of the meninges that surrounds the brain). However, recently subdural EEG recordings provided evidence that SDs occur in abundance in people with structural brain damage [1]. Indirect evidence was already provided earlier by functional magnetic resonance imaging (fMRI) [34] and a patient's symptom reports combined with fMRI [35] that SD also occurs in migraine with aura [2].

The slowest dynamics that can be accurately measured by EEG, i.e., the delta band, with frequencies about 0.5 to 4 Hz, has attracted modelling approaches much more than SD, which was doubted to occur in human brain until the first direct measurements were reported. It is interesting to compare the origin of slow time scales in such delta band models to our slow dynamics.

Models of the delta band essentially come in two types. On the one hand thalamo-cortical network and mean field models of HH neurons with fixed ion concentrations have been studied [36]. In this case, a slow time scale emerges because the cells are interconnected via synaptic connections using metabotropic receptors that are slow, because they act through second messengers. On the other hand, single neuron models with currents that are not contained in HH, namely a hyperpolarization-activated depolarizing current, 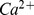-dependent sodium and potassium currents, and a persistent sodium current, were suggested. The interplay between these currents gives rise to oscillations at a frequency of about 2–3 Hz [37]. It is therefore hardly surprising that these currents, in particular the persistent sodium and the 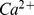-dependent sodium and potassium currents, have also been proposed to play an essential role in SD [30], [38]. Furthermore, bursting as another example of slow modulating dynamics was studied in a pure conductance-based model with a dendritic and an axo-somatic compartment [16]. Also metabotropic receptors as modeled by Bennett et al. [15] and other cellular processes at appropriately slow time scales may play a role and contribute to the repolarization.

In contrast to those approaches our results show that dynamics in a HH framework with time-dependent ion concentrations and buffer reservoirs already range from seconds to hours even with the original set of voltage-gated ion currents. Time scales from milliseconds (membrane dynamics) to seconds (ion dynamics) and even minutes to hours (ion exchange with reservoirs) can be directly computed from the model parameters (cf. Sect. Models). The interplay of membrane dynamics, ion dynamics and coupling to external reservoirs (glia or vasculature) naturally leads to dynamics typical of SLA and SD.

**Figure 11:**
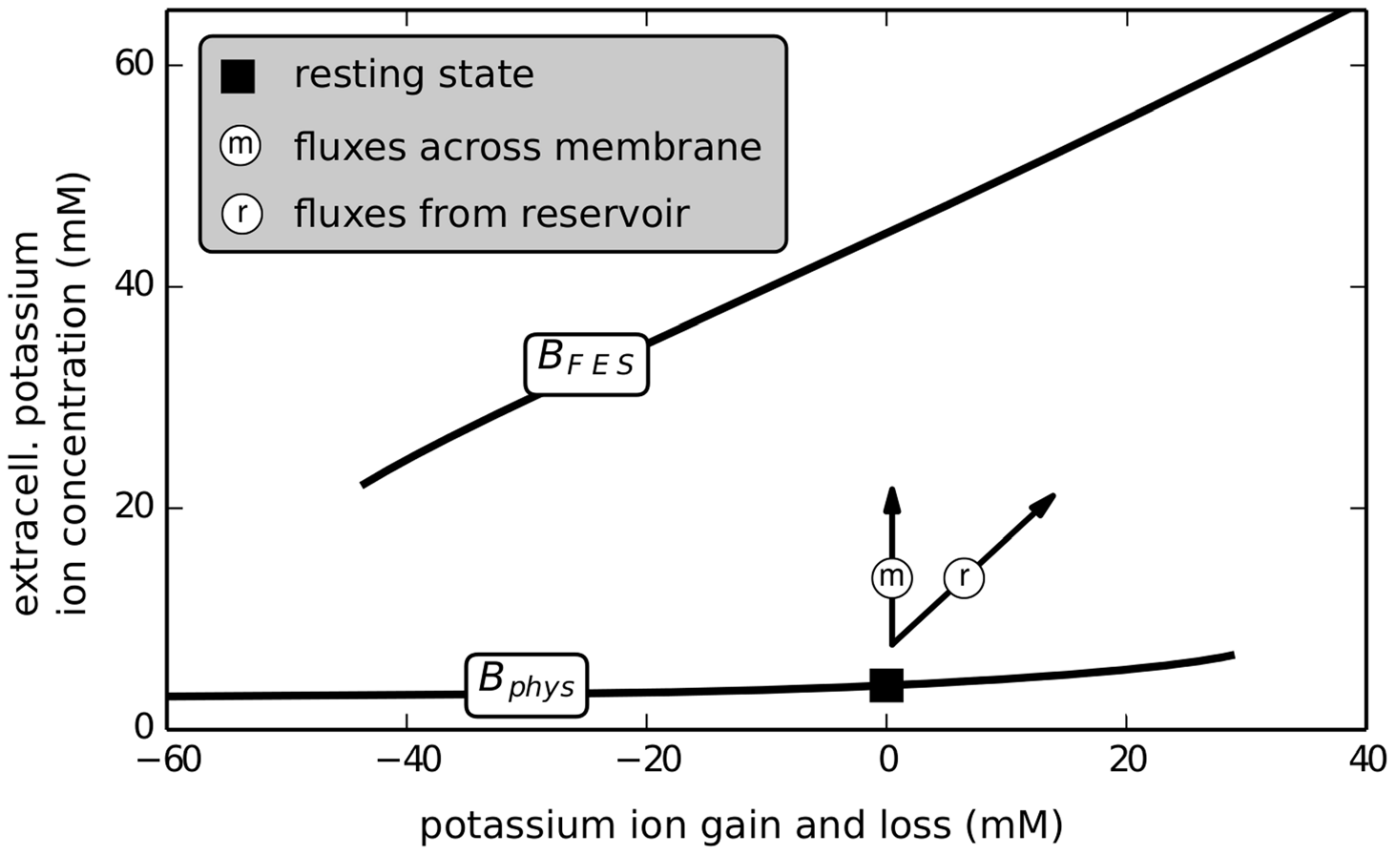
Bifurcation diagram. Fundamental bifurcation diagram in the slowest–scale dynamics, the potassium ion gain or loss through reservoirs (i.e., the bifurcation parameter). The unit of the bifurcation parameter was chosen such that it denotes the ion concentration with respect to the extracellular volume. The actual extracellular potassium concentration is the order parameter. Shown are the stable branches 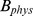 and 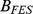 (see Sec. Results) and the directions (arrows) of two paths of ‘pure’ flux condition: fluxes exclusively across the membrane and fluxes exclusively from (or to) reservoirs. A horizontal path is caused by a particular mixture of these fluxes that induces potassium ion concentration changes exclusively to the intracellular compartment. Ionic excitability can be understood as a cyclic process in this diagram (see text).

In particular SD is explained by a bistability of neuronal ion dynamics that occurs in the absence of external reservoirs. The potassium gain or loss 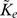 through reservoirs provided by an extracellular bath, the vasculature or the glial cells is identified as a bifurcation parameter whose essential importance was not realized in earlier studies (see Fig. 11). Using this bifurcation parameter and the extracellular potassium concentration as the order parameter, we obtain a folded fixed point curve with the two outer stable branches corresponding to states with normal physiological function, hence named physiological branch 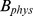, and to states being free-energy starved (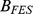).

The definition of the bifurcation parameter implies that exchange with ion reservoirs happens along the diagonal direction labelled by ‘r’. Membrane-mediated dynamics is in the vertical ‘m’ direction. In the full system where the ion exchange is a dynamical variable our unconventional choice of variables, i.e. modelling 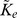 instead of 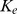, makes it obvious that the time scales of diagonal and vertical dynamics is separated by at least two orders of magnitude. Slow dynamics is along 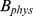 and 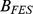, and the fast dynamics describes the jumps between these branches. We remark that dynamics along 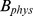 is slower than along 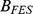, because the branch is almost horizontal which leads to a very small gradient driving the diffusive coupling. Similarly the release of buffered potassium from the glial cells is only weakly driven (cf. the discussion of buffering time scales in Sect. Model).

In the closed system sufficiently strong stimulations lead to the transition from the physiological resting state located on 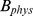 to FES. In the full system with dynamical ion exchange with the reservoirs, physiological conditions are restored after a large phase space excursion to the the before stable FES state. We refer to this process as ionic excitability. In contrast to the electrical excitability of the membrane potential this process involves large changes in the ion concentrations. The entire phase space excursion of this excitation process can be explained through the specific transits between and along 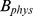 and 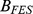.

We observe ion changes on three slow time scales. (i) Vertical transits between 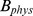 and 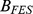 caused by transmembrane dynamics in the order of seconds. The time scale is determined by the volume-surface-area ratio and the membrane permeability to the ions. (ii) Diagonal dynamics along 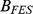 in the order of tens of seconds caused by contact to ion reservoirs. This time scale is determined by buffer time constants or vascular coupling strength. (iii) Dynamics on 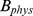 again caused by contact to ion reservoirs, but at the slower backward buffering time scale in the order of minutes to hours determined by the slower backward rate of the buffer [12]. During this long refractory phase of ionic excitability the spiking dynamics based on electrical excitability—separated by seven orders of magnitude—seems fully functional.

The right end of 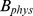 and the left end of 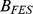 are marked by bifurcations that occur for an accordingly elevated or reduced potassium content. This is the first explanation of thresholds for local SD dynamics in terms of bifurcations. We remark, however, that for SD ignition the important question is not where 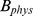 ends, but instead where the basin of attraction of 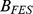 begins.

This new understanding of SD dynamics suggests a method to investigate the SD susceptibility of a given neuron model. One should consider the closed model without coupling to external reservoirs and check if shows the typical bistability between a physiological resting state and FES. We remark that unphysical so-called ‘fixed leak’ currents must be replaced by proper leak currents with associated leaking ions. Thresholds for the transition between 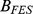 and 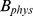 translate to thresholds for SD ignition and repolarization, i.e., recovery from FES in the full open model. Knowledge of the potassium reduction needed to reach the repolarization threshold and knowledge about the buffer capacity could then tell us if recovery from FES can be expected (such as in migraine with aura) or if the depolarization is terminal (such as in stroke).

Although our model does not contain all important processes involved in SD, our phase space explanation appears to be valid also for certain model extensions. For example, considering only diffusive regulation of potassium is physically inconsistent, but adding an analoguous regulation term for sodium turns out not to alter the dynamics qualitatively. Moreover osmosis-driven cell swelling—normally regarded as a key indicator of SD—is not included in our model, but can be added easily [13], [30], [39]. Unpublished results confirm that also with such cell swelling dynamics the fundamental bifurcation structure of Fig. 11 is preserved.

As a clinical application of our framework, we have linked a genetic defect, which affects the inactivation gate 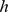 and which is present in a rare subtype of migraine with aura, to SD. Our simulations show that such mutations render neurons more vulnerable to SD [40]. The interesting point, however, is that on the level of the fast time scale the firing rate is decreased, which in a mean field approach (as done for the delta band) translates to decreased activity. This effect seemingly contradicts the increased SD susceptibility and hence illustrates the pitfalls in trying to neglect ion dynamics in the brain and to bridge the gap in time scales by population models.
